# The American Year Book of Medicine and Surgery

**Published:** 1900-11

**Authors:** 


THE AMERICAN YEAR BOOK OF MEDICINE AND SURGERY. Being a Yearly Digest of Scientific Progress and Authoritative Opinion in All Branches of Medicine and Surgery, drawn from Journals, Monographs, and Text-Books of the Leading American and Foreign Authors and Investigators. Collected and Arranged with Critical Editorial Comments by J. M. Baldy, M. D.; Chas. H. Burnett, M. D.; J. Chalmers DaCosta, M. D.; W. A. N. Dorland, M. D.; V. P. Gibney, M. D.; H. F. Hansell, M. D.; B. C. Hirst, M. D.; E. F. Ingals, M. D.; W. W. Keen, M. D.; H. G. Ohls, M. D.; Wendell Reber, M. D.; J. H. Waterman, M. D. Under the general editorial charge of Geo. M. Gould, M. D. Surgery, pp. 560; price, $3.75. Philadelphia: W. B. Saunders, 925 Walnut Street. 1900.

The plan of the publisher of dividing the Year-Book into two volumes is an improvement. The former single volume was unwieldy and hard to read without a rack or table on which to let the book rest. Then the specialist, in order to obtain the splendid review of his branches, had to purchase the entire volume, whereas, now he takes only the volume devoted to his specialty. The whole subject of medicine and surgery is reviewed in the two volumes, and the former high standard of the work is maintained.

